# Integrated analysis of MALDI-TOF MS and whole-genome sequencing for subtyping *Salmonella*

**DOI:** 10.3389/fmicb.2026.1782552

**Published:** 2026-03-09

**Authors:** Yuanshan Liu, Jianjian Qiao, Yue Xiao, Tao Zhang, Yiqin Wu

**Affiliations:** Yixing Center for Disease Control and Prevention, Wuxi, Jiangsu, China

**Keywords:** bacterial subtyping, machine learning, MALDI-TOF MS, *Salmonella*, whole-genome sequencing

## Abstract

Current subtyping methods are often restricted by labor intensity and high costs. To address this, this study integrated matrix-assisted laser desorption/ionization time-of-flight mass spectrometry (MALDI-TOF MS) with whole-genome sequencing (WGS) to characterize *Salmonella* isolates and investigate the correlation between spectral features and genomic data. Between 2023 and 2024, 96 *Salmonella* isolates from Yixing, Jiangsu Province, China, underwent serotyping, WGS, and MALDI-TOF MS profiling. Serotyping and Multilocus Sequence Typing (MLST) analysis resolved 25 serovars and 21 sequence types. Machine learning models based on spectral features achieved area under the curve (AUC) values exceeding 0.90 for *Salmonella typhimurium*, ST11, ST155, ST19, and ST34. Specific discriminatory mass peaks were identified, and their correlations with genomic annotations were investigated through peak-gene co-occurrence analysis. The findings indicate that discriminatory MALDI-TOF MS peaks can serve as statistical indicators for specific genomic features, reflecting underlying genomic differences. This study proposes a machine learning-based classification strategy that enables rapid analysis of MALDI-TOF MS spectra in routine diagnostics, thereby extending the application of mass spectrometry in *Salmonella* subtyping. This strategy functions as a high-throughput pre-filter to concentrate WGS efforts on high-risk clones for accelerated outbreak investigation.

## 1 Introduction

Non-typhoidal *Salmonella* is a leading cause of foodborne gastroenteritis globally (Stanaway et al., 2019; Havelaar et al., 2015). Despite the genus’s diversity, only a few dominant serovars, such as *Salmonella typhimurium* and *S. enteritidis*, account for the majority of human infections. These lineages representing high-risk clones are responsible for the majority of severe human infections globally (Pulford et al., 2021; Sia et al., 2024). Therefore, rapid differentiation of these epidemiologically relevant lineages is critical for outbreak response.

Historically, *Salmonella* surveillance has relied on the Kaufmann-White serotyping scheme (Kitching, 1945). While widely used, this phenotypic method is labor-intensive and dependent on costly antisera (Banerji et al., 2020). Over the past decades, molecular methods such as MLST (Stepan et al., 2011) and Pulsed-Field Gel Electrophoresis (PFGE) (Thong et al., 2002) have improved discriminatory power. Recently, Whole-Genome Sequencing (WGS) has emerged as the gold standard for phylogenetics due to its ultimate resolution (Nadon et al., 2017; Akinyemi et al., 2023). However, the resource-intensive nature of sequencing and complex bioinformatics limits its utility for routine screening in frontline laboratories. Therefore, there is an urgent need for a rapid, cost-effective tool to achieve precise *Salmonella* subtyping.

Matrix-assisted laser desorption/ionization time-of-flight mass spectrometry (MALDI-TOF MS) has become a standard method for species-level identification (Oviano and Rodriguez-Sanchez, 2021). Although historically considered insufficient for subtyping, recent studies indicate that subtle spectral differences can discriminate some *Salmonella* serovars (Ren et al., 2025). However, prior research has predominantly focused on spectral classification, while the statistical associations between discriminatory peaks and lineage-specific genomic features remain uncharacterized. This disconnect confines MALDI-TOF MS to simple phenotypic clustering, failing to reveal the specific genetic determinants that define epidemiologically important lineages, thereby reducing its resolution for outbreak investigation.

To address this limitation, this study established a machine learning strategy that not only performs rapid subtyping but also maps discriminatory mass peaks to specific genomic gene sets through peak-gene co-occurrence analysis. Through a peak-gene co-occurrence analysis of 96 isolates from diverse hosts, we demonstrate that specific spectral peaks serve as signature features for epidemiologically relevant lineages. This method thus acts as a rapid screening tool to guide targeted WGS of high-risk isolates, thereby optimizing outbreak surveillance.

## 2 Materials and methods

### 2.1 Bacterial isolates

**Figure 1:**
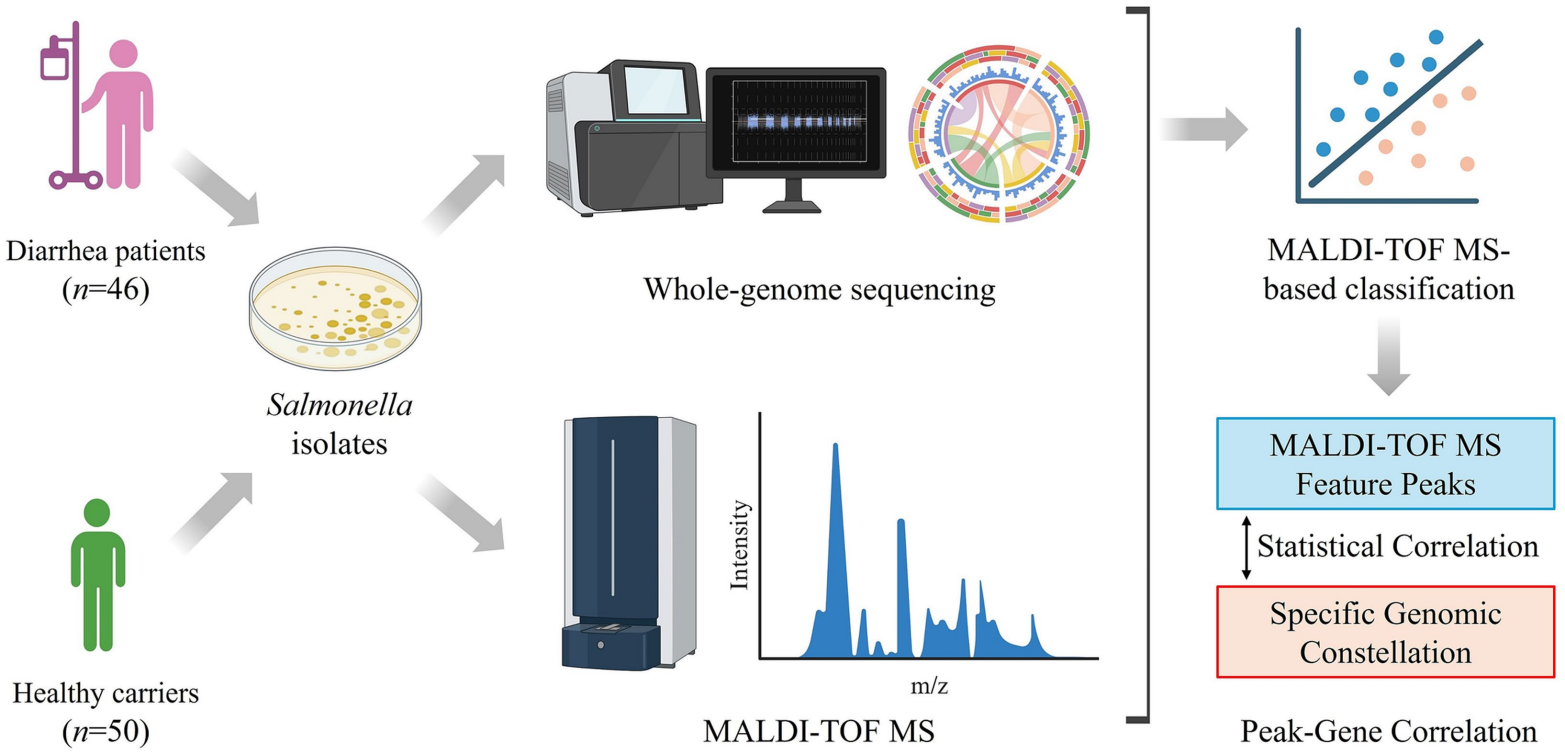
Schematic overview of the study workflow integrating MALDI-TOF MS and whole-genome sequencing for *Salmonella* analysis.

From 2023 to 2024, a total of 96 *Salmonella* isolates were recovered from stool specimens collected in Yixing, Jiangsu Province, China, including 46 isolates from outpatients with foodborne diarrhea and 50 isolates from routine fecal screening of healthy carriers in medical institutions (Figure 1). All isolates were preserved in the laboratory of the Yixing Center for Disease Control and Prevention (Yixing, Jiangsu Province, China). This study was approved by the Ethics Committee of the Yixing Center for Disease Control and Prevention (2023YX-jy-1).

### 2.2 Instruments and reagents

Bacterial cultures were incubated in incubators (Thermo Fisher Scientific, USA). Species identification and MALDI-TOF MS spectral acquisition were performed on an EXS1600 automated microbial mass spectrometry system equipped with the manufacturer’s data analysis software (Zhongyuan Huiji, China).

*Salmonella* chromogenic agar and blood agar plates (CHROMagar, France) were used for isolation and subculture. A commercial *Salmonella* diagnostic antisera kit (Statens Serum Institut, Denmark) was used for serotyping. A ready-to-use microbial sample pretreatment kit and *α*-cyano-4-hydroxycinnamic acid (CHCA) matrix solution for MALDI-TOF MS (Zhongyuan Huiji, China) were used according to the manufacturer’s instructions. A 70% (v/v) formic acid solution of analytical grade was used for on-target extraction.

### 2.3 Culture conditions, identification and serotyping

Isolation and culture of presumptive *Salmonella* were performed according to the National Food Safety Standard (GB 4789.4-2024) and the technical guidelines of the National Pathogen Surveillance Network of China. Colonies with morphology compatible with *Salmonella* on primary enrichment plates were streaked onto *Salmonella* chromogenic agar plates for purification. After incubation, well-isolated colonies were subjected to species identification using the EXS1600 MALDI-TOF MS system following the manufacturer’s standard protocol for microbial detection.

Isolates identified as *Salmonella* spp. by MALDI-TOF MS were cultured onto blood agar plates. Serotyping was then performed using the commercial *Salmonella* diagnostic antisera kit according to the manufacturer’s instructions. O and H antigen profiles were interpreted with reference to the White-Kauffmann-Le Minor antigen scheme, and serovars were assigned to each isolate accordingly.

### 2.4 MALDI-TOF MS sample preparation and instrumental conditions

For MALDI-TOF MS, fresh colonies were obtained from blood agar plates after overnight incubation. For each isolate, a single fresh colony was randomly selected, and approximately one colony volume was picked using a sterile 1 μL inoculation loop. The biomass was evenly smeared onto a designated spot of a polished stainless-steel MALDI target plate and was allowed to air-dry completely at room temperature. Subsequently, 1 μL of 70% formic acid was pipetted onto the dried smear to lyse the bacterial cells, and the spot was again allowed to air-dry completely. After drying, 1 μL of CHCA matrix solution was dispensed onto the same spot to cover the sample. The matrix-analyte mixture was allowed to air-dry to form a homogeneous crystalline layer before analysis. For each isolate, two technical replicate spectra were acquired and averaged after alignment to generate a single consensus spectrum per isolate. Spectra were acquired in positive linear ion mode using the manufacturer’s standard settings for microbial identification. The mass-to-charge (*m/z*) range was set to approximately 2,000–20,000 Da. A laser frequency of 60 Hz was applied, and for each spectrum 200 laser shots were automatically accumulated by the instrument software to generate a final profile spectrum. Instrumental calibration was performed daily using *Escherichia coli* ATCC 25922 as an external standard, which was prepared via the ethanol/formic acid extraction method according to the manufacturer’s protocol. Calibration was validated by the presence of seven characteristic peaks at m/z 3637.80, 4365.30, 5096.80, 5381.40, 6255.40, 7274.50, and 10300.10.

### 2.5 Whole-genome sequencing and bioinformatic analyses

Genomic DNA was extracted from the bacterial isolates using the BG-Flex-48 automated nucleic acid extraction system with the Bacterial Genomic DNA Extraction Kit (Model: NEG-48B-L, Bojie Medical Technology, Shanghai, China). The concentration and purity of the DNA were quantified using a Qubit 3.0 Fluorometer and agarose gel electrophoresis. Only samples with a total DNA amount ≥100 ng were used for library construction.

Sequencing libraries were prepared using the Whole Genome DNA Library Prep Kit (Model: SMSJ-CX-606, Bojie Medical Technology) following the manufacturer’s instructions. Briefly, genomic DNA was fragmented, end-repaired, A-tailed, and ligated with sequencing adapters. Target fragments (~350 bp) were selected using magnetic beads and enriched by PCR. The library quality was assessed using an Agilent 4150 Bioanalyzer and quantified by qPCR (>2 nM).

The libraries were sequenced on the DNBSEQ-T7 platform (MGI Tech Co., Ltd.) with a paired-end 150 bp (PE150) strategy, generating at least 1 Gb of raw data per sample. Raw reads were processed using fastp (v0.23.2) to remove adapters and low-quality sequences (Q30 > 85%). The clean reads provided an average sequencing depth of approximately 200×. Assembly was performed using SPAdes (v3.15.5). Species identification and contamination removal were conducted using Kraken2 (v2.1.2) to ensure only *Salmonella* scaffolds were retained for downstream analysis. The raw and processed sequencing data were deposited in the National Microbiology Data Center (NMDC) under project accession NMDC10019769.^1^

Genome assemblies were annotated using Prokka (v1.13) to identify coding DNA sequences and predicted proteins (Seemann, 2014). Multilocus sequence typing (MLST) was performed using the MLST software (v2.23.0) based on the seven-housekeeping gene scheme (*aroC*, *dnaN*, *hemD*, *hisD*, *purE*, *sucA*, and *thrA*) for *Salmonella enterica* (Jolley and Maiden, 2010). Antimicrobial resistance (AMR) genes and virulence factors were identified using ABRicate (v1.0.1) by querying the CARD and VFDB databases, respectively. To ensure high-confidence identification, thresholds for both minimum DNA identity and minimum coverage were set to 80%. The reported gene counts include comprehensive profiles encompassing both acquired elements and intrinsic chromosomal genes. A minimum-spanning tree based on MLST allelic profiles was constructed using the NetworkX library in Python 3.10. For phylogenomic analysis, all 96 *Salmonella* genomes were aligned using REALPHY (v1.12) to generate core-genome alignments (Bertels et al., 2014). The resulting phylogenetic tree was visualized, annotated and coloured using the Evolview web server.

### 2.6 MALDI-TOF MS data preprocessing and peak extraction

Raw MALDI-TOF MS spectra exported from the EXS1600 system were preprocessed using custom Python 3.10 scripts. For each isolate, the profile spectrum intensities were interpolated onto a common *m/z* grid to construct an intensity matrix.

To account for differences in total signal intensity, total ion current (TIC) normalization was applied to each spectrum. The TIC-normalized spectra were then smoothed using a Savitzky–Golay filter with a window length of 5 data points and a polynomial order of 2. After smoothing, peak calibration was performed across spectra.

Peak detection was performed using a relative intensity threshold of 2% of the maximum intensity per spectrum to distinguish meaningful signals from background noise. Detected peaks from different isolates were aligned using an *m/z* tolerance of 2.0 *m/z* units. Peaks whose *m/z* values fell within the same tolerance window across isolates were considered to represent the same feature and were merged into a single consensus peak.

### 2.7 Machine learning model construction and evaluation

MALDI-derived peak features were matched to the corresponding genomic data using isolate identifiers, and a unified feature matrix was constructed. MALDI-TOF MS features with a detection frequency of at least 3% across the 96 isolates were retained for subsequent analyses. Three groups of One-vs-Rest (OvR) binary classification tasks were defined: (i) sample source (diarrhea patients vs. healthy carriers), (ii) major serotypes, and (iii) major sequence types.

For these tasks, a sparse logistic regression model with L1 regularization (Lasso) was constructed using the Scikit-learn library. L1 regularization was selected to enforce model sparsity, which effectively performs feature selection by shrinking the coefficients of non-informative spectral peaks to zero. This approach mitigates the high-dimensionality of mass spectrometry data and enhances the biological interpretability of the identified markers. To prioritize the identification of positive cases, a threshold moving strategy was implemented, lowering the decision cutoff to 0.2 (Freeman and Moisen, 2008). This lower threshold was selected to prioritize sensitivity, ensuring that potentially high-risk isolates are flagged for further verification in a public health surveillance context. To address class imbalance without altering the original data structure, algorithmic class weighting (class weight = ‘balanced’) was applied. Model performance was evaluated using stratified 5-fold cross-validation. The discriminative ability of the model was quantified using the Area Under the Curve (AUC), sensitivity and specificity.

A One-vs-Rest (OvR) strategy was employed to identify lineage-specific markers (Rifkin and Klautau, 2004). For each binary classification task, the probability $P(y_{i}=1|x_{i})$ that sample *i* belongs to the target lineage is modeled using the logistic function (Equation 1):


$$P(y_{i}=1|x_{i})=\frac{1}{1+e^{-(\beta_{0}+\sum_{j=1}^{m}\beta_{j}x_{\mathrm{ij}})}}\;$$ (1)

where $x_{i}$ represents the peak feature vector of *i*-th sample, *x_ij_* represents the intensity of the *j*-th peak feature for the *i*-th sample, *m* is the total number of spectral features, $\beta_{0}$ is the intercept term, and β represents the vector of weight coefficients.

To address class imbalance, class weighting was applied during optimization (Friedman et al., 2010). The model parameters were estimated by minimizing the weighted L1 regularized negative log-likelihood function (Equation 2):


$$\min_{\beta_{0},\beta }\{\begin{array}{l} -\sum_{i=1}^{n}[w_{1}y_{i}\log(p_{i})+w_{0}(1-y_{i})\log(1-p_{i})]\\ +\lambda \sum_{j=1}^{m}|\beta_{j}| \end{array}\}$$ (2)

where $p_{i}$ denotes the predicted probability from logistic function (Equation 1), *n* is the number of training samples, and $w_{1}$ and $w_{0}$ represent the class weights for the positive and negative classes, respectively. These weights are defined as $w_{k}=\frac{N_{\text{total}}}{n_{\text{classes}}\times N_{k}}$ using the total number of samples *N_total_*, number of classes *n_classes_*, and the sample count *N_k_* for class *k* (*k* ∈ {0, 1}) to balance the contribution of each class to the loss function by inversely proportional weighting. The term $\lambda \sum_{j=1}^{m}|\beta_{j}|$ is the L1 penalty that enforces sparsity to select discriminatory features.

Discriminatory MS peaks were defined as features with non-zero coefficients ( $\beta_{j}\neq 0$ ) in the optimized model. The Importance Index $I_{j}$ for each peak was calculated as the normalized absolute coefficient (Equation 3):


$$I_{j}=\frac{|\beta_{j}|}{\sum_{l=1}^{m}|\beta_{l}|}\times 100\%$$ (3)

where the denominator represents the sum of the absolute values of all *m* coefficients, and *l* serves as the summation index across all features.

### 2.8 Integrated analysis of proteomic and genomic features

To elucidate the genomic associations of the spectral features, we integrated MALDI-TOF MS data with WGS results. First, hierarchical clustering of isolates was performed based on MLST allelic profiles using Hamming distance, and proteomic profiles were visualized via heatmaps using log10-transformed intensities of top discriminative peaks.

Subsequently, a statistical association analysis was conducted between MS peaks and gene presence/absence patterns. To align the quantitative spectral data with the binary nature of genomic gene profiles (presence/absence), peak intensities were binarized using an adaptive K-Means clustering (*k* = 2) approach. The correlation strength was quantified using the Phi coefficient (*ϕ*), and significance was assessed via Chi-squared tests with Benjamini-Hochberg (FDR) correction (*p* < 0.05) (Noble, 2009). To focus on biologically robust associations, only significant peak-gene co-occurrence patterns (*ϕ* > 0.3) were visualized using bubble plots, where genes were sorted based on their dominant peak correlations, excluding hypothetical proteins.

The complete analysis pipeline is available at https://github.com/liuyuanshan017/YiXing-Salmonella/tree/main.

### 2.9 Statistical analysis

All data processing, statistical analyses, and visualizations were performed using Python (version 3.10). Continuous variables were presented as means ± standard deviations (SD). For the high-dimensional peak-gene association analysis, *p* values were adjusted using the Benjamini-Hochberg false discovery rate (FDR) method to control for multiple comparisons. FDR-adjusted *p* value < 0.05 was considered statistically significant.

## 3 Results

### 3.1 Serotype and sequence-type distribution of *Salmonella* isolates

A total of 96 *Salmonella* isolates were included in the analysis, comprising 46 isolates from diarrhea patients and 50 isolates from healthy carriers. Serotyping assigned these isolates to 25 distinct serovars. *Salmonella typhimurium* was predominant, accounting for 42.7% (41/96) of isolates. *Salmonella enteritidis* and *S. London* each accounted for 9.4% (9/96), and *S. Derby* accounted for 5.2% (5/96), with the remaining serovars each comprising fewer than five isolates.

MLST analysis grouped the 96 isolates into 21 sequence types (STs). ST19 was the most frequent ST (26.0%, 25/96), followed by ST34 (19.8%, 19/96), ST11 (12.5%, 12/96), ST155 (8.3%, 8/96) and ST358 (5.2%, 5/96). Each of the remaining STs was represented by fewer than five isolates.

### 3.2 Phylogenetic structure and distribution of resistance and virulence genes

**Figure 2:**
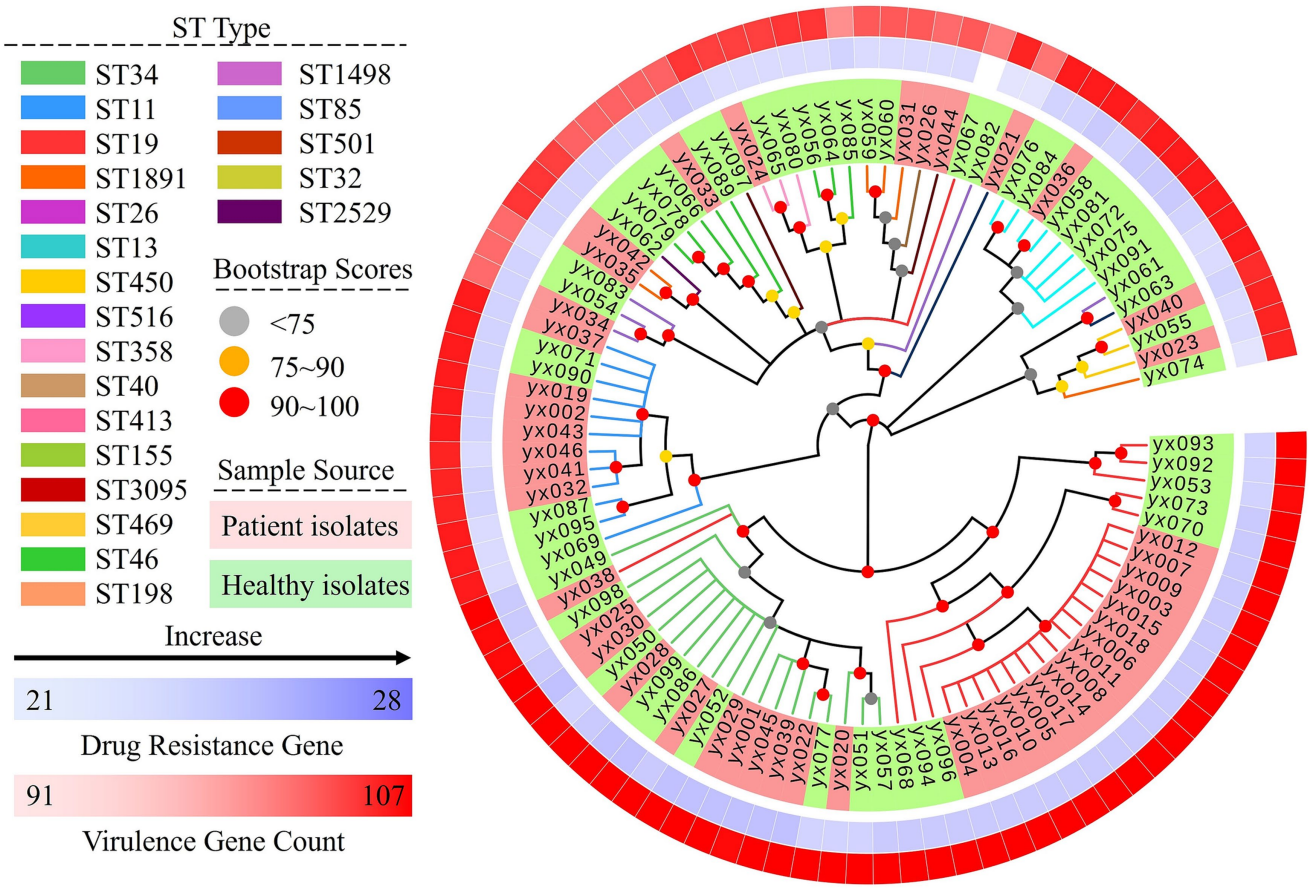
Phylogenetic relationships and genomic characteristics of 96 *Salmonella* isolates.

The phylogenetic tree resolved the 21 STs into several major lineages (Figure 2). ST19, ST34, ST11 and ST155 each formed distinct clusters. Within the ST19 cluster, 68.0% (17/25) of isolates originated from diarrhea patients, whereas 12.5% (1/8) of isolates within the ST155 cluster originated from diarrhea patients. For the ST34 and ST11 clusters, the proportions of patient-derived isolates were 52.6% (10/19) and 58.3% (7/12), respectively.

On average, each isolate harbored 26.2 ± 1.1 antimicrobial resistance genes. Intrinsic resistance genes accounted for the majority of detections (25.6 ± 0.7), whereas acquired mobile resistance genes were infrequent (0.7 ± 0.6) (Supplementary Table S1) (Alcock et al., 2020). Virulence gene counts averaged 103.5 ± 4.2 per isolate. These were predominantly core virulence genes, including SPI-1 (28.9 ± 0.8), SPI-2 (31.0 ± 0.1), fimbrial clusters (16.1 ± 1.8), and other core genes (24.1 ± 1.1). Variable prophage-associated genes were lineage-specific and limited in abundance (3.4 ± 1.5) (Supplementary Table S2) (Liu et al., 2019).

### 3.3 Identification of discriminatory MALDI-TOF MS features across different subtypes

**Figure 3:**
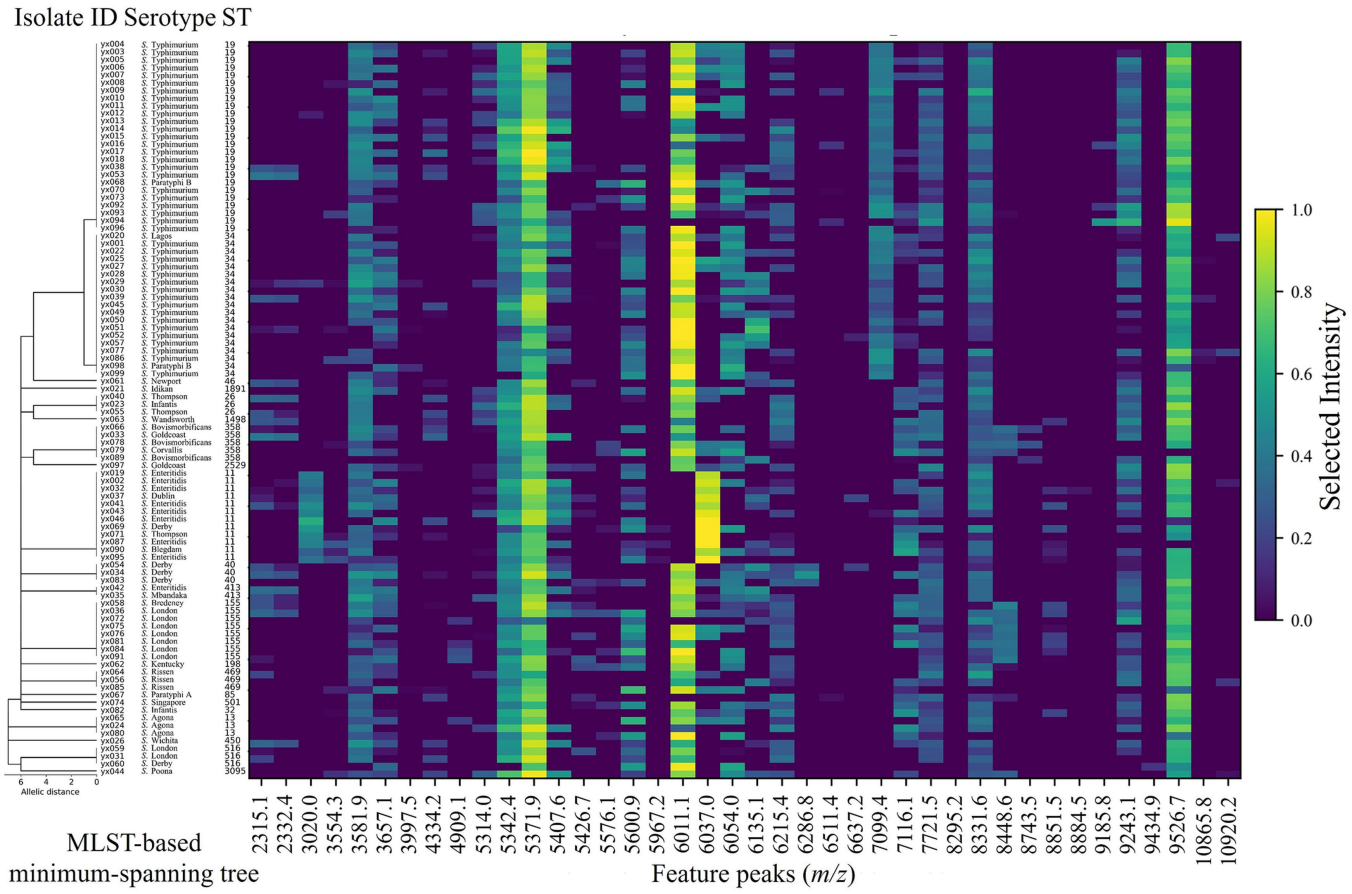
Heatmap of discriminative MALDI-TOF MS peak intensities ordered by MLST clustering.

Based on the multi-task classification analysis, global peaks were ranked by their comprehensive discriminatory importance. Consequently, the top 40 peaks were identified as the primary discriminatory features. As illustrated in the heatmap (Figure 3), MALDI-TOF MS spectral fingerprints were highly conserved within isolates of the same ST, whereas distinct peak patterns differentiated separate STs. Notably, certain peaks manifested as continuous high-intensity bands exclusive to specific STs. For instance, prominent ions at *m/z* 3020.0 and 6037.0 were characteristic of ST11. Similarly, *m/z* 7099.4 was associated with ST19 and ST34, while *m/z* 6286.8 was unique to ST40. The peak at *m/z* 8448.6 was restricted to a minor subset of STs, including ST358 and ST155.

### 3.4 Performance evaluation of MALDI-TOF MS-based classification models

As shown in Table 1, the source classification model yielded an AUC of 0.970 with a sensitivity of 97.8%. The relatively lower specificity (74.0%) was due to the implementation of a sensitivity-prioritized threshold. For serotype identification, the model performed well for the dominant serotype, *S. typhimurium* (AUC 0.945, sensitivity 95.1%, specificity 89.1%). Conversely, predictive accuracy for rare serotypes (such as *S.* London, *S. enteritidis*, and *S.* Derby) was moderate and lower than that for *S. typhimurium*. The sequence type (ST) prediction model achieved the highest predictive efficacy at the genotypic level. Both ST11 and ST155 achieved optimal classification, with AUCs of 1.000 and 100% sensitivity (specificity: 98.8 and 93.2%, respectively), indicating distinct MALDI-TOF MS spectral signatures. ST19 and ST34 also showed strong discriminatory potential (AUCs: 0.962 and 0.926; sensitivity: 96.0 and 89.5%), although specificities were comparatively lower (74.6 and 70.1%). In contrast, ST358 exhibited moderate performance (AUC 0.862, sensitivity 60.0%, specificity 91.2%), potentially constrained by the limited positive sample size.

**Table 1:** Performance evaluation of MALDI-TOF MS-based classification models for *Salmonella* source tracking, serotyping, and sequence typing.

Task	Class	No. of isolates (Pos/Neg)	AUC	Sensitivity	Specificity
Source	Diarrhea patients vs. healthy carriers	46/50	0.970	0.978	0.740
Serotype	*S. typhimurium*	41/55	0.945	0.951	0.891
	*S.* London	9/87	0.862	0.889	0.747
	*S. enteritidis*	9/87	0.786	0.778	0.862
	*S.* Derby	5/91	0.809	0.600	0.945
Sequence type	ST19	25/71	0.962	0.960	0.746
	ST34	19/77	0.926	0.895	0.701
	ST11	12/84	1.000	1.000	0.988
	ST155	8/88	1.000	1.000	0.932
	ST358	5/91	0.862	0.600	0.912

### 3.5 Correlation between MALDI-TOF MS feature peaks and genomic annotations

**Figure 4:**
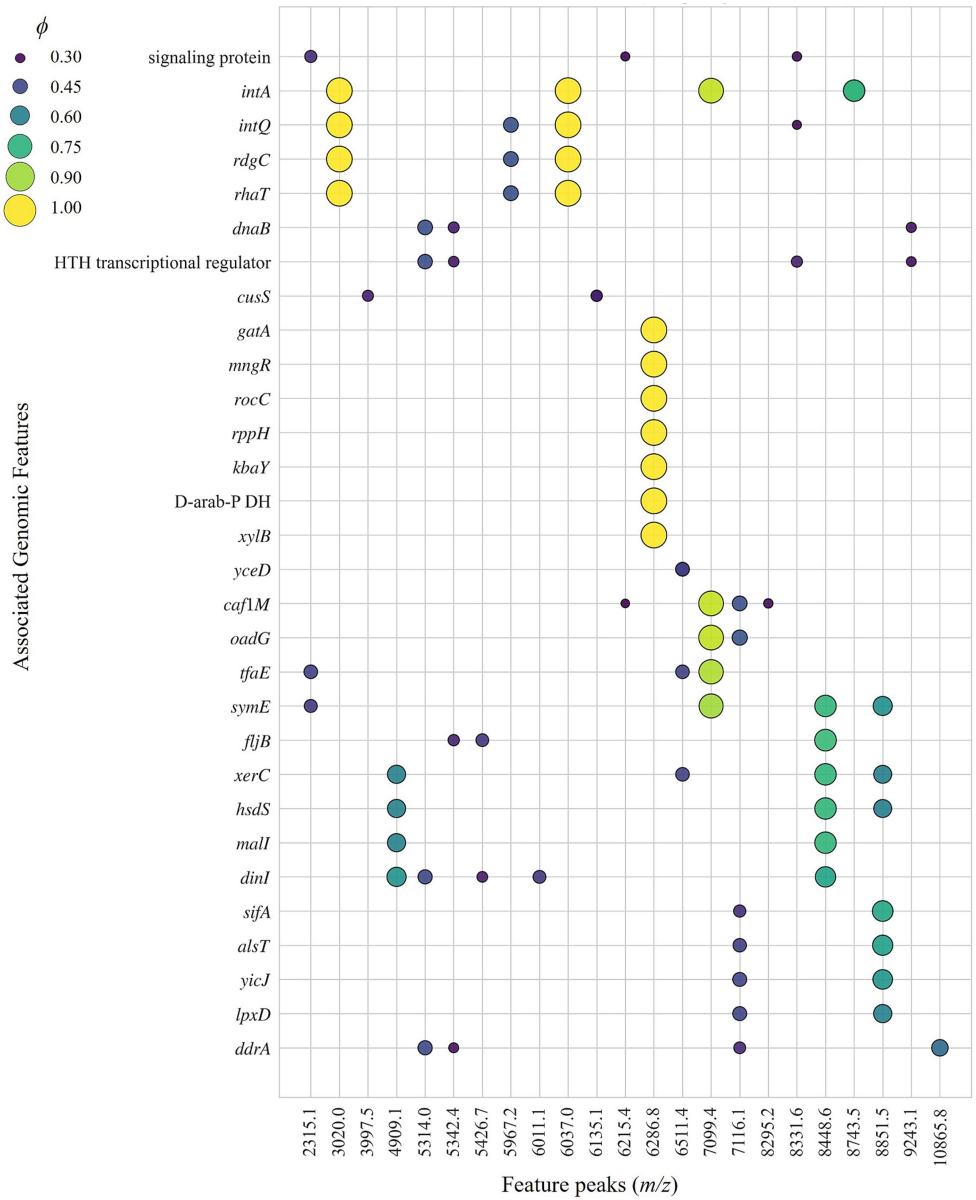
Correlation analysis between MALDI-TOF MS feature peaks and WGS-derived genomic annotations.

The importance of feature peaks derived from different machine learning models was ranked to identify critical markers. The top three features for each classification model, along with their relative importance percentages, are shown in Table 2. To investigate the genomic associations of these features, a correlation analysis was performed between binarized MALDI-TOF MS data and WGS annotations using the Phi coefficient (*ϕ*). Significant associations (FDR-adjusted *p* < 0.05 and *ϕ* > 0.3) were visualized as a bubble plot (Figure 4).

**Table 2:** Key discriminatory spectral feature peaks and importance percentages for different classification tasks.

Task	Class	Top1 Peak (*m/z*, %)	Top2 Peak (*m/z*, %)	Top3 Peak (*m/z*, %)
Source	Diarrhea patients vs. healthy carriers	5407.6 (14.9%)	8331.6 (12.8%)	3581.9 (6.7%)
Serotype	*S. typhimurium*	7099.4 (42.8%)	10920.2 (12.7%)	5576.1 (8.9%)
	*S.* London	4909.1 (13.6%)	6215.4 (9.8%)	5426.7 (9.0%)
	*S. enteritidis*	3020.0 (17.1%)	6637.2 (8.3%)	2332.4 (7.2%)
	*S.* Derby	6286.8 (40.5%)	5967.2 (14.5%)	9434.9 (13.7%)
Sequence type (ST)	ST19	7099.4 (28.1%)	10865.8 (10.7%)	5371.9 (9.2%)
	ST34	6011.1 (11.4%)	10865.8 (11.2%)	6135.1 (7.5%)
	ST11	6037.0 (59.2%)	3020.0 (36.9%)	8295.2 (2.9%)
	ST155	8448.6 (44.5%)	4909.1 (18.7%)	8851.5 (16.9%)
	ST358	8743.5 (26.3%)	9526.7 (13.0%)	7721.5 (9.7%)

Specifically, ions at *m/z* 3020.0 (36.9% importance) and 6037.0 (59.2%) emerged as the most critical features for ST11. These two peaks exhibited high co-occurrence and strong correlation within the samples. Furthermore, they showed significant associations with the prophage integrase IntA (*intA*), the defective protein IntQ (*intQ*), the L-rhamnose-proton symporter (*rhaT*), and the recombination-associated protein RdgC (*rdgC*).

For *S.* Derby, the peak at *m/z* 6286.8 was identified as a defining feature (40.5%). This peak correlated significantly with D-arabitol-phosphate dehydrogenase (D-arab-P DH), xylulose kinase (*xylB*), mannosyl-D-glycerate transport/metabolism system repressor MngR (*mngR*), the PTS system galactitol-specific EIIA/B/C component (*gatA/B/C*), and RNA pyrophosphohydrolase (*rppH*).

The ion at *m/z* 7099.4 also served as a key classification metric, demonstrating high importance for both *S. typhimurium* (42.8%) and ST19 (28.1%). This peak showed significant linkages to *intA*, prophage tail fiber assembly protein TfaE (*tfaE*), endoribonuclease SymE (*symE*), chaperone protein caf1M (*caf1M*), oxaloacetate decarboxylase gamma chain (*oadG*).

Regarding ST155, the peak at *m/z* 8448.6 was a predominant feature (44.5%) and was significantly associated with toxic protein SymE (*symE*), phase 2 flagellin (*fljB*), tyrosine recombinase XerC (*xerC*), type-1 restriction enzyme EcoKI specificity protein (*hsdS*), maltose regulon regulatory protein MalI (*malI*), and DNA damage-inducible protein I (*dinI*). Additionally, the peak at *m/z* 4909.1 was identified as a significant marker for both ST155 (18.7%) and *S.* London (13.6%), sharing significant correlations with *xerC*, *hsdS*, *malI*, and *dinI*.

## 4 Discussion

In this study, we integrated MALDI-TOF MS spectral profiling with WGS data to characterize *Salmonella* isolates from Yixing, Jiangsu Province, China. The epidemiological data revealed the predominance of *S. typhimurium*, represented by ST19 and ST34, consistent with the major epidemic lineages currently prevalent in China (Sun et al., 2020, 2025). Although the dominant serovars and sequence types are concordant with those reported in neighboring cities (Yu et al., 2024), variation in their relative abundance reveals regional characteristics of the local *Salmonella* population.

In recent studies, incorporating MALDI-TOF MS data into machine learning has enabled the distinction of closely related subtypes. Mangmee et al. (2020) used MALDI-TOF mass spectrometry typing for predominant serovars of non-typhoidal *Salmonella* in a Thai broiler industry. Gao et al. (2023) rapidly identified *Salmonella* serovars Enteritidis and Typhimurium using whole cell MALDI-TOF MS coupled with multivariate analysis and artificial intelligence. The machine learning model based on MALDI-TOF MS spectroscopy used in this study can distinguish major *Salmonella* serovars and sequence types. The classifiers for dominant subtypes, including *S. typhimurium*, ST19, ST34, ST11, and ST155, achieved high diagnostic accuracy with AUC values exceeding 0.90. However, for subtypes with few positive isolates, such as *S.* Derby and ST358, the predictive sensitivity was reduced. Furthermore, the developed machine learning model demonstrated great discriminatory performance in distinguishing *Salmonella* between two sample sources: diarrheal patients and healthy carriers, suggesting spectral differences among these sources.

Based on the constructed machine learning models, a set of MALDI-TOF MS feature peaks relevant to various classification tasks was identified. Distinct *Salmonella* subtypes frequently possess unique gene sets, thereby exhibiting characteristics different from other subtypes. Therefore, correlating key spectral features with WGS data revealed the statistical associations between discriminatory mass peaks and subtype-specific gene sets.

The machine learning models indicated that *m/z* 6037.0 and *m/z* 3020.0 are decisive features for ST11, and *m/z* 3020.0 is the most important feature peak for *S. enteritidis*. ST11 represents the major global lineage of *S. enteritidis* (Achtman et al., 2012), and Dieckmann and Malorny (2011) previously reported the signal at *m/z* 6,036 as a specific marker for *S. enteritidis*. *m/z* 6037.0 and *m/z* 3020.0 showed significant correlation with *intA*, *intQ*, *rdgC*, and *rhaT*, representing the unique gene set of the corresponding *Salmonella* subtypes.

For *S.* Derby, the prominent feature peak at *m/z* 6286.8 showed significant correlations with multiple metabolic genes (*gatA, mngR, rocC, rppH, kbaY, xylB,* D-arab-P DH), indicating that these genes constitute a unique gene set of the *S.* Derby lineage. Hayward et al. (2015) demonstrated that *S.* Derby possesses a distinct metabolic phenotype characterized by specific substrate utilization patterns. Sevellec et al. (2018) indicated that such metabolic characteristics contribute to lineage-specific adaptations to particular hosts, such as swine and poultry.

For *S. typhimurium* and ST19, the characteristic peak at *m/z* 7099.4 exhibited significant correlations with *intA*, *caf1M*, *oadG*, *tfaE*, and *symE*. Among these, *intA*, *tfaE*, and *symE* are associated with prophage-encoded virulence determinants, while *caf1M* contributes to adhesive structures, consistent with the high pathogenicity of this lineage. Wahl et al. (2019) highlighted that prophages act as a driving force in reshaping the genome, frequently contributing virulence factors through lysogenic conversion. Acuña et al. (2020) described *symE* as part of an SOS-responsive Type I toxin-antitoxin system, characterized by endoribonuclease activity that regulates bacterial survival under stress conditions.

Additionally, *m/z* 8448.6 and *m/z* 4909.1 exhibited simultaneous significant associations with genes such as *xerC*, *hsdS*, *malI*, and *dinI*. This indicates that multiple distinct feature peaks can collectively correlate with the same genomic markers. Consequently, discriminatory MALDI-TOF MS peaks often map to the unique genomic sets of corresponding subtypes. Future studies could expand sample sizes to leverage these gene-spectrum statistical relationships or construct machine learning models, thereby enabling the prediction of specific genes directly from MALDI-TOF MS spectra.

However, this study still has some limitations. The sample size (*n* = 96) and geographical restriction to a single region may limit the diversity of the training dataset. As a result, the classification models showed reduced sensitivity for rare serovars due to class imbalance. Additionally, consistent with interpreting these features as statistical indicators of lineage, the precise molecular identities of the discriminatory peaks remain uncharacterized. While correlations with genomic profiles were established, definitive proteomic identification via LC–MS/MS was not performed to determine the biological origin of these signals. Future research should prioritize expanding dataset diversity and could employ proteomic analysis to elucidate the molecular basis of these spectral markers.

## 5 Conclusion

This study demonstrated the efficacy of integrating MALDI-TOF MS spectral profiling with WGS for rapid characterization of *Salmonella* lineages. Machine learning analysis confirms that spectral phenotypes exhibit phylogenetic resolution comparable to that of MLST. Correlating spectral features with genomic annotations, it shows that the predictive power of MALDI-TOF MS is derived from the inherent phylogenetic structure of *Salmonella*. While current WGS approaches remain the gold standard for phylogenetics, these findings indicate that MALDI-TOF MS can serve as an efficient screening tool for rapid lineage identification in routine diagnostics. This study enables a high-throughput screening method where MALDI-TOF MS serves as a pre-filter, concentrating WGS efforts on high-risk clones to accelerate outbreak investigation.

## Data Availability

The names of the repository/repositories and accession number(s) can be found at: https://nmdc.cn/resource/genomics/project/detail/NMDC10019769.

## Footnotes

1
https://nmdc.cn/resource/genomics/project/detail/NMDC10019769
