# Supplementary material for: Integrated analysis of MALDI-TOF MS and whole-genome sequencing for subtyping Salmonella
Source: Front Microbiol. 2026 Mar 9;17:1782552. doi: 10.3389/fmicb.2026.1782552 (PMC13006217; doi:10.3389/fmicb.2026.1782552)
Supplement: Supplementary file 1 [file Table_1.docx]

Supplementary Material

Table S1. Distribution of resistance genes for different categories

| Category | Group | N | Intrinsic Genes | Acquired Genes | Total Genes |
| --- | --- | --- | --- | --- | --- |
| Total | All Isolates | 96 | 25.6±0.7 | 0.7±0.6 | 26.2±1.1 |
| Source | Patient | 46 | 25.7±0.5 | 0.9±0.6 | 26.5±1.0 |
|  | Healthy | 50 | 25.5±0.9 | 0.4±0.6 | 25.9±1.2 |
| Serotype | *S*. Typhimurium | 41 | 26.0±0.0 | 1.1±0.6 | 27.1±0.6 |
|  | *S*. London | 9 | 25.8±0.4 | 0.4±0.5 | 26.2±0.8 |
|  | *S*. Enteritidis | 9 | 25.1±0.3 | 0.6±0.5 | 25.7±0.5 |
|  | *S*. Derby | 5 | 25.0±0.0 | 0.4±0.5 | 25.4±0.5 |
| ST | ST19 | 25 | 26.0±0.0 | 0.9±0.3 | 26.9±0.3 |
|  | ST34 | 19 | 26.0±0.0 | 1.2±0.9 | 27.2±0.9 |
|  | ST11 | 12 | 25.0±0.0 | 0.7±0.5 | 25.7±0.5 |
|  | ST155 | 8 | 26.0±0.0 | 0.5±0.5 | 26.5±0.5 |
|  | ST358 | 5 | 26.0±0.0 | 0.2±0.4 | 26.2±0.4 |

**Note: Intrinsic genes**: *AAC(6')-Iaa, acrB, acrD, bacA, baeR, cpxA, CRP, emrA, emrB, emrR, Escherichia_coli_acrA, Escherichia_coli_ampH, golS, H-NS, kdpE, marA, mdsA, mdsB, mdsC, mdtB, mdtC, mdtK, msbA, sdiA, tolC, yojI*. **Acquired Genes**: *TEM-1, tet(B)*.

Table S2. Distribution of virulence genes for different categories

| Category | Group | N | SPI-1 Genes | SPI-2 Genes | Fimbriae Genes | Variable Genes | Other Core Genes | Total Genes |
| --- | --- | --- | --- | --- | --- | --- | --- | --- |
| Total | All Isolates | 96 | 28.9±0.8 | 31.0±0.1 | 16.1±1.8 | 3.4±1.5 | 24.1±1.1 | 103.5±4.2 |
| Source | Patient | 46 | 29.0±0.2 | 31.0±0.0 | 16.3±1.7 | 3.7±1.5 | 24.3±1.0 | 104.3±3.9 |
|  | Healthy | 50 | 28.8±1.1 | 31.0±0.2 | 16.0±1.9 | 3.1±1.4 | 23.8±1.2 | 102.7±4.4 |
| Serotype | *S*. Typhimurium | 41 | 29.0±0.0 | 31.0±0.0 | 17.0±0.0 | 4.9±0.3 | 25.0±0.0 | 106.9±0.3 |
|  | *S*. London | 9 | 29.0±0.0 | 31.0±0.0 | 15.9±2.2 | 2.6±0.9 | 24.0±0.0 | 102.4±3.1 |
|  | *S*. Enteritidis | 9 | 29.0±0.0 | 31.0±0.0 | 16.9±0.3 | 2.6±0.5 | 23.9±0.3 | 103.3±1.0 |
|  | *S*. Derby | 5 | 29.0±0.0 | 31.0±0.0 | 13.0±2.2 | 2.0±0.7 | 22.2±1.6 | 97.2±3.9 |
| ST | ST19 | 25 | 29.0±0.0 | 31.0±0.0 | 17.0±0.0 | 4.9±0.3 | 25.0±0.0 | 106.9±0.3 |
|  | ST34 | 19 | 29.0±0.0 | 31.0±0.0 | 17.0±0.0 | 4.9±0.3 | 25.0±0.0 | 106.9±0.3 |
|  | ST11 | 12 | 29.0±0.0 | 31.0±0.0 | 17.0±0.0 | 2.8±0.5 | 24.0±0.0 | 103.8±0.5 |
|  | ST155 | 8 | 29.0±0.0 | 31.0±0.0 | 17.0±0.0 | 2.9±0.4 | 23.9±0.4 | 103.8±0.7 |
|  | ST358 | 5 | 29.0±0.0 | 31.0±0.0 | 12.0±0.0 | 2.0±0.0 | 22.0±0.0 | 96.0±0.0 |

**Note:** **SPI-1 genes**: *avrA, invA, invB, invC, invE, invF, invG, invH, invI, invJ, orgA, orgB, orgC, prgH, prgI, prgJ, prgK, sicA, sicP, sipA/sspA, sipB/sspB, sipC/sspC, sipD, spaO, spaP, spaQ, spaR, spaS, sptP*. **SPI-2 genes**: *spiC/ssaB, ssaC-V, sscA-B, sseA-G, sseJ, sseL*. **Fimbriae genes**: *csgA-G, fimC-I, lpfA-E*. **Variable genes**: *gogB, sspH2, sseK1, sseK2, sseI/srfH*. **Other core genes**: *entA, entB, fepC, fepG, mgtB, mgtC, misL, mig-14, ompA, pipB, pipB2, ratB, sifA, sifB, sinH, slrP, sodCI, sopA, sopB/sigD, sopD, sopD2, sopE2, steA, steB, steC*.
